# Ultra-high resolution photon-counting CT of the lung after lung transplantation: should we go for optimal image quality or reduced radiation dose?

**DOI:** 10.1016/j.jhlto.2026.100547

**Published:** 2026-04-11

**Authors:** Adriana Dubbeldam, Valerie Van Ballaer, Johny Verschakelen, Emanuele Muscogiuri, Joke Binst, Lesley Cockmartin, Hilde Bosmans, Walter Coudyzer, Kwinten Torfs, Emanuele Di Dedda, Johan Coolen, Walter de Wever

**Affiliations:** a Deparment of Radiology, UZ Leuven, Belgium; b Deparment of Medical Physics, UZ Leuven, Belgium

**Keywords:** Computed tomography, Lung, Lung transplantation, Photon-counting CT, Energy-integrating detector CT, Ultra-high resolution

## Abstract

**Objectives:**

As an often-young population with regular CT follow-up, lung transplant (LTX) patients could benefit from both high spatial resolution and lower radiation dose. We compared the image quality of ultra-high-resolution photon-counting CT (UHR-PCCT) with high-resolution energy-integrating-detector CT (HR-EIDCT) at both a normal (IQ100) and a reduced radiation dose (IQ40) in patients after LTX.

**Methods:**

Hundred patients post-LTX, with an UHR-PCCT (0.4 mm) between Dec-2021 and Aug-2022, and prior HR-EIDCT (1.0 mm) <14 months apart, were included. In 49 patients, PCCT-IQ100 images were compared with previous HR-EIDCT; in 51 patients, PCCT-IQ40 images were compared with previous HR-EIDCT. Image quality was scored using a 5-point Liker scale (−2 to +2) for 7 relevant entities (peripheral airways, micronodules, reticulations, pleural thickening, consolidation, ground glass opacities (GGO), and air-trapping) plus an overall quality impression score.

**Results:**

For PCCT-IQ100, the median visual grading analysis (VGA) score was significantly better than EIDCT for all structures/abnormalities (median < 0; *p* < 0.005) and for most structures/abnormalities in PCCT-IQ40 (*p* < 0.023). There was no perceived diagnostic impact in either group (median = 2; *p* ≥ 0.039). Mean dose length product (DLP) was reduced by 33% in PCCT-IQ100 and 73% in PCCT-IQ40 compared to EIDCT (*p* < 0.0001).

**Conclusions:**

A significant dose reduction was achieved using the PCCT-IQ40 protocol. Although image quality was best in PCCT-IQ100, image quality in PCCT-IQ40 was non-inferior to EIDCT. Therefore, a significant radiation dose reduction is feasible without compromising image quality.

**Clinical relevance statement:**

PCCT offers improved image quality while reducing radiation dose significantly.

For lung transplant patients, who are often younger and require lifelong, regular follow-up with CT, both improved image quality and lowered radiation dose would be very beneficial.

**Key Points:**

- LTX patients require long-term follow-up with high-resolution imaging to detect often subtle abnormalities and would benefit from both improved image quality and radiation dose reduction.

- Perceived image quality was significantly higher in IQ100-PCCT than EIDCT; a significant further radiation dose reduction was feasible (IQ40-PCCT) without compromising image quality compared to EIDCT.

## Background

Together with pulmonary function tests, high-resolution CT scans (HRCT) are considered the backbone of diagnosis and follow-up of many diffuse lung diseases, including interstitial lung disease (ILD), cystic fibrosis, and emphysema. For many years, developments in HRCT have been slowly but constantly progressing, with the development of multidetector energy-integrating-detector-CT (EIDCT), dual-energy imaging and several postprocessing software upgrades.^1^ This has improved the quality of CT-images, lowered radiation dose, and speeded up acquisition time. At the current time, some consider EIDCT to be near its full potential, with further dose reduction limited by the occurrence of image noise and artifacts; similarly, a higher image resolution comes with the disadvantage of increased radiation dose.^2^

With the approval of the first clinical photon-counting detector CT (PCCT) in 2021, a new technology has become available that detects and counts individual photons, converting each X-ray photon into an electrical signal. This leads to a better differentiation between signal and electronic noise, allowing for ultra-high resolution scanning and providing information on spectral resolution.^3^, ^4^ PCCT is of special interest in thoracic imaging, where ultra-high-resolution may influence the way we see the primary anatomic unit of the lung, the secondary pulmonary lobule, currently just out of reach compared to microscopic imaging.^5^, ^6^, ^7^ On the other hand, reaching a radiation dose close to a 2-view chest X-ray has been the goal for many years, especially with ongoing plans for lung cancer screening.^8^

Although PCCT offers both improved resolution at a reduced radiation dose, further reduction of the radiation dose comes at the price of reduced clinical image quality, and vice versa. Which optimization route to choose (optimal image quality or reduced radiation dose) will depend on the diagnostic purpose of the study. In our study, we focused on the implementation of an optimal ultra-high-resolution photon-counting CT (UHR-PCCT) protocol specifically in the follow-up of patients after lung transplantation.

Patients in follow-up after lung transplantation require lifelong follow-up with pulmonary function tests and bronchoscopy. Regular imaging with HRCT is also re--ed by the International Society for Heart and Lung Transplantation (ISHLT),^9^ if available and in the form of a yearly HRCT including an expiratory scan to detect late or chronic complications such as immunosuppression-related infection, posttransplant lymphoproliferative disease, and chronic lung allograft dysfunction (CLAD).^10^ CLAD, also referred to as “chronic rejection,” is considered a major life-limiting complication seen in around 41% of transplant recipients.^10^ In CLAD, specific subtypes have been described with either restrictive pulmonary function (restrictive allograft syndrome or RAS), or obstructive pulmonary function decline (bronchiolitis obliterans syndrome or BOS).^10^, ^11^, ^12^ The typical pattern on CT for RAS includes reticular and fibrotic changes, often in the upper lobes, with pleural thickening suggesting pleuroparenchymal fibro-elastosis.^13^, ^14^ Subtle reticular findings can precede the clinical diagnosis based on pulmonary function testing. In BOS, abnormalities on CT are more conspicuous and often appear during a later stage of the disease, with signs of progressive airway disease such as bronchial dilatation, bronchial wall thickening, and especially air-trapping.^10^, ^15^

To detect these changes early in the disease course, especially in RAS,^14^ imaging at ultra-high-resolution with PCCT could prove valuable. On the other hand, this serial imaging with X-ray and CT (pre- and post-transplant) can lead to a significant cumulative radiation dose, with increased risk of radiation-related malignancies.^16^, ^17^

In this study, we evaluated the image quality of high-resolution chest CT on conventional energy-integrating-detector CT (HR-EIDCT) compared to UHR-PCCT, at a normal radiation dose level (IQ100) and at a reduced radiation dose level (IQ40), in patients after lung transplantation.

## Materials and methods

### Patient selection

**Figure 1:**
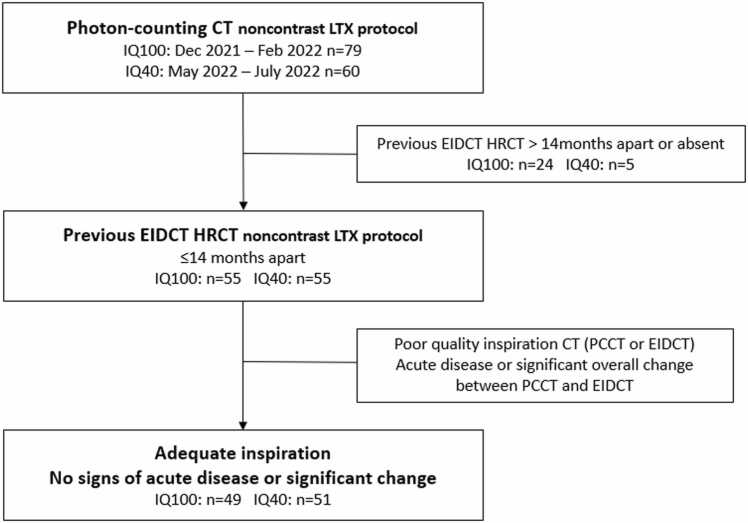
*Selection criteria.* Abbreviations: LTX, lung transplantation; IQ, image quality level; EIDCT, energy-integrating-detector CT; HRCT, high-resolution CT; PCCT, photon-counting CT.

Approval of the Ethics Committee was obtained for all photon-counting scans, including informed consent by every patient prior to scanning. In September 2021, our hospital installed their first commercial photon-counting CT scanner. Patient recruitment ran between December 2021 and August 2022, with a 3-month period of scanning at the initial optimized dose settings (IQ100) (Dec-Feb 2022) and a 3-month period of scanning at lower radiation dose settings (IQ40) (May-Jul 2022). Of the 139 patients scanned between Dec-21 and Aug-22, 110 patients had a previous HR-EIDCT at our institution no older than 14 months compared to the PCCT scanning date. During the imaging evaluation, we excluded another 10 patients in which either the PCCT or EIDCT was compromised (e.g., no adequate inspiration) or showed a significant overall change between scans (e.g., diffuse inflammatory or infectious abnormalities) (Figure 1).

### Imaging acquisition and processing

For the follow-up of lung transplant patients, our institution uses a specific post lung transplant scanning protocol, comprising of a high-resolution acquisition at optimal inspiration and at expiration. Data acquisition parameters are shown in Table 1.

**Table 1:** Image Acquisition Parameters

	PCCT	EIDCT
Collimation	120×0.2 mm	64×0.625 mm
		80×0.5 mm
		128×0.6 mm
		192×0.6 mm
Slice thickness	0.4 mm	1.0 mm
Increment	0.2 mm	0.5-0.7 mm
kVp-settings	120 kVp	110-120 kVp
Pitch	0.85	1.2
Rotation time	0.5 sec	0.5 sec

Abbreviations: EIDCT, energy-integrating-detector CT; kVp, kilo voltage peak; PCCT, photon-counting CT.

PCCT-imaging was performed on the NAEOTOM Alpha (Siemens Healthineers). Exposure setting are determined by Care Dose4D (tube current modulation). The reconstruction matrix (512×512, 768×768, or 1024×1024) was automatically determined by the scanner based on the field-of-view, in correlation with patient size and scout view. Reconstruction kernels were Br40 for soft tissue and BL56-60 for lung tissue. No iterative reconstruction was used. Forty-nine patients were scanned using initial dose (IQ100), while 51 were scanned using lower radiation dose settings (IQ40).

Previous EIDCT was performed on one of 6 EIDCT-scanners in our department (Siemens Somatom Force (17 patients); Siemens Somatom Definition Flash (28 patients); Siemens Definition Edge (7 patients); Toshiba Acquilion One (28 patients); Philips Ingenuity (20 patients).

Reconstruction kernels were a soft kernel for soft tissue and a sharp kernel for lung tissue and varied between scanners.

Radiation doses were recorded in the local dose management system (DOSE, Qualum, Belgium) and sent to the PACS system. All CT-scanners are monitored by the medical physics team and are optimized for image quality level.

### Image analysis and scoring

In the setting of lung transplantation and its well-known long-term complications (as described in the introduction), the following relevant structures/abnormalities were selected for scoring: peripheral airways, micronodules, reticulations, pleural thickening, consolidation, ground glass opacities (GGO) and air-trapping. Also, overall image quality was compared for each inspiratory scan. IQ100 and IQ40 were randomly mixed to reduce scoring bias.

As mentioned, patients were excluded when either PCCT or EIDCT inspiration was inadequately performed, or when a significant overall change occurred between EIDCT and PCCT (e.g., diffuse inflammation or infection) that made it impossible to compare image quality accurately (correlated with consensus agreement). However, since it is likely small changes will occur in the scanning interval of up to 14 months, careful attention and consensus agreement was applied to exclude the specific abnormalities that changed or were not adequately comparable between scans (e.g., poor expiration), while non-affected structures/abnormalities were still scored by the readers.

Structures/abnormalities were scored as present or absent, and consensus was obtained for discrepancies. PCCT vs EIDCT was then score side-by-side through a visual grading analysis (VGA) using a 5-point Likert scale, ranging from −2 to +2, by 3 experienced thoracic radiologists and 1 radiologist-in-training (reader R1-R4):

-2: decreased visibility on PCCT with perceived impact on diagnostic interpretation.

-1: decreased visibility on PCCT without perceived impact.

0: similar visibility between PCCT and EIDCT.

+1: improved visibility on PCCT without perceived impact.

+2: improved visibility on PCCT with perceived impact on diagnostic interpretation.

The specific experience with thoracic Radiology of each reader was 35 years (R4), 8 years (R2), 2 years (R3) and 0 years (the last being a radiology resident in the last year of training to become a thoracic radiologist - R1).

### Statistical analysis

Continuous data (e.g., patient demographics) is represented using mean, standard deviation and range. Comparisons were performed using unpaired Student *T*-test.

Ordinal data (e.g., VGA-scores using Likert-scale) is represented as median and interquartile range. Comparisons were performed using a 1-sample Wilcoxon signed rank test with hypothesized median values of 0 (similar image quality PCCT vs EIDCT) and +2 (improved visibility on PCCT with perceived impact on diagnostic interpretation).

Statistical significance level was conducted as a 2-tailed significance level of 5% (α-level 0.05) and applying the Bonferroni correction for multiple comparisons. Analysis was done using SPSS statistical software (v.28.0 IBM).

Inter-observer agreement was calculated using an intraclass correlation coefficient with a 2-way random and absolute agreement definition.

### Global noise analysis

An assessment of noise was made by calculating global noise level (GNL) in the sharp kernel reconstructions of all scans.^18^, ^19^ This GNL analysis was performed with an in-house built tool using the method of Christianson.^18^ GNL was calculated in the soft tissue (0-170 HU), the fatty tissue (−300-0 HU) and the lung tissue (<−600 HU). GNL for each scan was defined as the average GNL of 10 equidistant slices. Mean GNL was calculated for all PCCT-IQ100, all PCCT-IQ40 and EIDCT cases.

## Results

### Patient characteristics

Patient demographics are shown in Table 2. The study group included 48 women and 52 men. Mean age at imaging was 57 years (range 25-78 years), while the mean age at transplantation was 50 years (range 20-66 years). Water-equivalent-diameter, as a representation of patient attenuation properties, was not significantly different between IQ100 and IQ40 (26.1 ± 3.4 in IQ100 vs 26.8 ± 3.6 in IQ40; *p* < 0.0001). Scanning time between PCCT and EIDCT was on average 9 months (range 1-14 months), with an interval of 0-3 months in 17% of patients, 3-6 months in 10%; 6-9 months in 7%; 9-12 months in 60%, and 12-14 months in 6%. Scanning time after lung transplantation was on average 75 months, representing mostly patients in long-term follow-up (91%). Out of 100 patients, 7 patients were deceased before the submission of this paper, with severe infection as a main reason (6 patients, including 1 COVID-infection). Based on the analysis of the HRCT reports (PCCT and EIDCT), many patients developed air-trapping (69%), representing the development of BOS in many of these patients (other reasons include bronchial anastomosis narrowing or postinfectious sequelae). Thirty-four patients had linear opacities on CT (34%). Of these, 7 patients had mild reticulations in the upper lobes, suggestive of early RAS. Five patients showed a significant upper lobe fibrosis, compatible with a radiological diagnosis of RAS.

**Table 2:** Patient Demographics

	IQ100		IQ40		PCCT	
Number of patients	49		51		100	
Gender (female/male)	23 F/26 M		25 F/26 M		48 F/52 M	
WED	26.1 ± 3.4		26.8 ± 3.6		26.5 ± 3.5	
Age	53 ± 14	(25-78)	60 ± 11	(27-74)	57	(25-78)
Age at transplantation	47 ± 14	(20-66)	52 ± 12	(21-66)	50	(20-66)
Time between scans	8±4	(1-12)	9 ± 4	(1-12)	9	(1-12)
Scan time after LTX	75 ± 68	(3-320)	95 ± 60	(6-255)	85	(3-320)
Reason for LTX						
EMPH/COPD	18	37%	30	59%	48	48%
ILD	12	24%	8	16%	20	20%
CF	13	27%	8	16%	21	21%
Other	6	12%	5	10%	11	11%
Air-trapping/BOS in report	32	65%	37	73%	69	69%
Deceased/alive	4/49	8%	3/51	6%	7/100	7%

Continuous variables are displayed as mean ± standard deviation (range).

Abbreviations: CF, cystic fibrosis; EMPH/COPD, emphysema or chronic obstructive pulmonary disease; ILD, interstitial lung disease; IQ, image quality level; LTX, lung transplantation; WED, water-equivalent diameter.

### Image parameters, radiation dose, and global noise analysis

Reconstruction matrix was 512 × 512 in EIDCT. For PCCT, the reconstruction matrix varied depending on patient size, field-of-view, and scout view and varied between 512 × 512 (52%), 768 × 768 (13%), and 1024 × 1024 (35% of cases). Pixel size was 0.750.08 (range 0.54-0.90) for EIDCT and 0.60 ± 0.20 (range 0.36-0.95) for PCCT (*p* < 0.0001).

Radiation dose and noise levels are summarized in Table 3. The radiation doses were significantly reduced on PCCT, especially at the IQ40-setting. DLP was reduced by 33% in PCCT-IQ100 compared to EIDCT, and CTDIvol was reduced by 15%. Switching to the PCCT-IQ40 protocol reduced radiation dose by 58% (DLP; 57% for CTDIvol) compared to PCCT-IQ100 and by 73% (DLP; 64% for CTDIvol) compared to EIDCT.

**Table 3:** Radiation Dose and Global Noise Level Comparison

	EIDCT	PCCT	
DLP, *mGy.cm*	662.7 ± 334.5	323.2 ± 202.4	*p* < 0.0001
CTDI vol, *mGy*	8.0 ± 4.4	4.8 ± 2.6	*p* < 0.0001
GNL soft tissue, *HU*	98.5 ± 28.8	93.2 ± 9.9	*p* = 0.1027
GNL lung tissue, *HU*	96.5 ± 29.7	93.9 ± 15.9	*p* = 0.5777
GNL fat tissue, *HU*	95.0 ± 27.2	92.3 ± 8.4	*p* = 0.2265
	**IQ100**	**IQ40**	
DLP, *mGy.cm*	446.4 ± 146.8	188.7 ± 65.1	*p* < 0.0001
CTDI vol, *mGy*	6.8 ± 2.3	2.9 ± 1.0	*p* < 0.0001
GNL soft tissue, *HU*	89.7 ± 10.6	96.5 ± 7.9	*p* < 0.0001
GNL lung tissue, *HU*	87.0 ± 13.1	100.4 ± 15.7	*p* < 0.0001
GNL fat tissue, *HU*	88.6 ± 8.4	95.7 ± 6.8	*p* < 0.0001

Continuous variables are displayed as mean ± standard deviation (range)

Abbreviations: CTDIvol, volume computed tomography dose index; DLP, dose length product; GNL, global noise level; HU, Hounsfield units; IQ, image quality level.

GNL in the lung tissue was significantly lower for PCCT-IQ100 than for PCCT-IQ40 (GNL = 87.0±13.1 HU) vs GNL = 100.4 ± 15.7HU) (*p* < 0.0001) and for PCCT-IQ100 compared to EIDCT (GNL = 96.5 ± 29.7HU) (*p*<0.05). The same trend is seen in the soft tissue, with GNL, respectively, 89.7 ± 10.6HU and 96.5 ± 7.9HU) (*p* < 0.0001) for PCCT IQ100 and IQ40. However, the GNL for PCCT-IQ40 is at a comparable level to EIDCT with GNL = 98.5 ± 28.8HU (*p* > 0.05). For the fatty tissue, the same results are found with GNL, respectively, 88.6 ± 8.4HU and 95.7 ± 6.8HU (*p* < 0.0001) for IQ100 and IQ40 and 95.0 ± 27.2HU (*p* > 0.05) for EIDCT. (Figure * - Supplemental Material).

### Qualitative assessment - per structure

*IQ100:* In all the structures/abnormalities that were scored, the median VGA score for all 4 readers together was significantly better in PCCT-IQ100 than EIDCT (median>0; *p* < 0.005). The only exception was GGO (*p* = 0.053) because of the low number of patients with GGO (*N* = 7). Median VGA score for all readers was also unequal to +2 (median = 2; *p* *≥* 0.016). A median VGA-score of 0 was most frequently given (61%), followed by +1 (39%). A median VGA-score of −1 was given in 1%, all of which in the category of air-trapping. A median score of +2 or −2 was never given.

**Figure 2:**
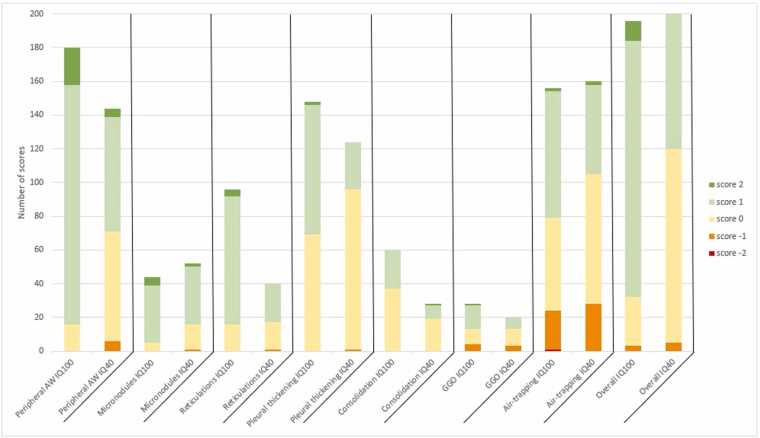
*Frequency histogram of absolute scores for IQ100 and IQ40*, A score of +2 is most frequently found for peripheral airways and overall image quality. Negative scores were mostly seen for air-trapping. A score of −2 was very rare. Abbreviations: AW, airways; GGO, ground glass opacities. * Red: median VGA-score of −2; orange: score of −1; yellow: score of 0; light green: score of +1; dark green: median VGA-score of +2.

Since the median VGA-scores range mostly from 0 to +1, looking at the absolute times a certain score was given provides more information on the distribution of the scores (Figure 2) (Table * - Supplemental Material).

**Figure 3:**
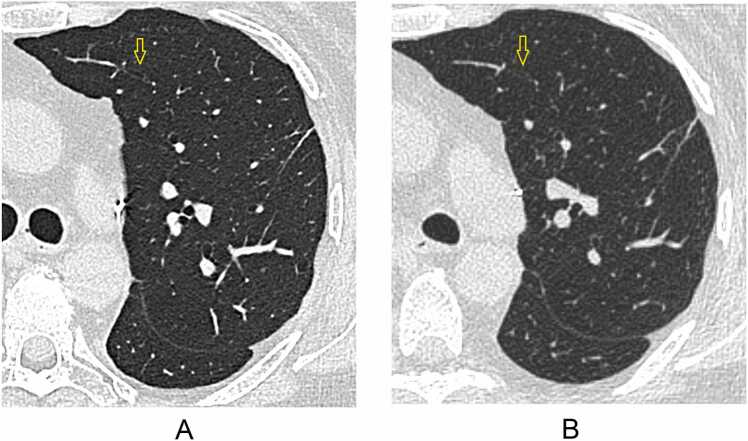
*Example of VGA +2 for peripheral airways in IQ100*. Patient received a score of +2 for peripheral airways by multiple readers, suggesting increased visibility of the structure with perceived diagnostic impact. (A) UHR-PCCT shows sharp delineation of an anterior peripheral airway (arrow); (B) the same airway is difficult to detect on HR-EIDCT (arrow).

A score of +1 was the most frequent score in peripheral airways (79%), micronodules (77%), and reticulations (79%); it was also the most frequent score in pleural thickening (52%), GGO (50%), and air-trapping (48%), albeit it close the frequency of a 0-score (47%, 32%, and 35%, respectively). A score of 0 was most frequent in consolidations (62%). A score of +2 was occasionally seen in all structures/abnormalities except consolidation and usually in <10% of scores, except for peripheral airways (12%) and micronodules (11%) (Figure 3). The highest cumulative scores (≥+1) were seen for peripheral airways (91%), micronodules (89%), and reticulations (83%).

**Figure 4:**
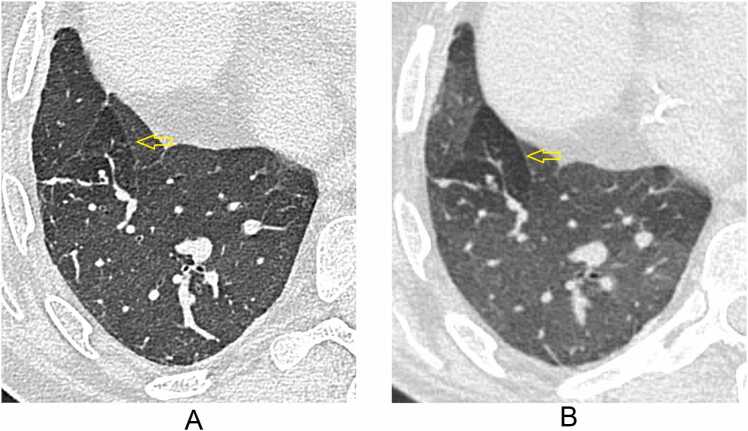
*Example of VGA −1 for air-trapping in IQ100*. Patient received a score of −1 for air-trapping by multiple readers, suggesting reduced visibility without perceive diagnostic impact. (A) UHR-PCCT shows a low image contrast between the area of air-trapping (arrow) and adjacent normal lung tissue; (B) HR-EIDCT shows clear image contrast between the same areas of air-trapping (arrow) and adjacent normal lung tissue.

A score of −1 was only seen in GGO (14%) and air-trapping (15%) (Figure 4). A score of −2 was only seen for air-trapping and only once (1%).

*IQ40:* In PCCT-IQ40 vs EIDCT, the median VGA score was significantly better in PCCT (median > 0; *p* < 0.023) for most structures/abnormalities, except consolidations (*p* = 0.102) and GGO (*p* = 0.180), both with a low number of patients (CSL *N* = 7; GGO *N* = 5). The median VGA score was significantly different from +2 (median = 2; *p* *≥* 0.039).

A median VGA-score of 0 was most frequently given (88%), followed by +1 (12%). A median VGA-score of −1 was given in 1 patient in the category of air-trapping. A median score of +2 or −2 was never given. Absolute numbers are show in Figure 2.

**Figure 5:**
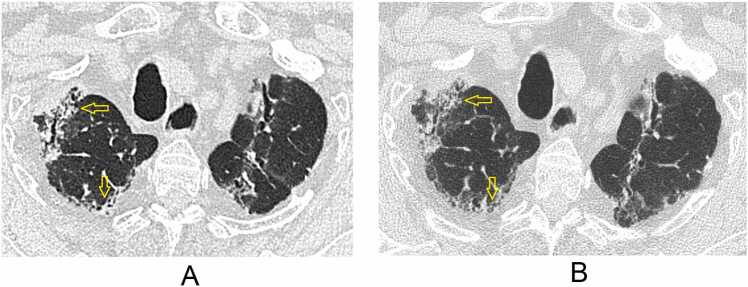
*Example of VGA +1 for reticulations in IQ40*. Patient with RAS received a score of +1 for reticulations by multiple readers, suggesting improved visibility without perceived diagnostic impact. (A) UHR-PCCT shows sharp delineation of fine reticulations in the right upper lobe (arrows); (B) HR-EIDCT shows more unsharp/hazy appearance of the same area of fine reticulations (arrows), although the diagnosis of reticulations and RAS could still be made. Abbreviations: RAS, restrictive allograft syndrome; UHR-PCCT, ultra-high-resolution photon-counting CT; HR-EIDCT, high-resolution energy-integrating-detector CT. The most experienced reader was R4 with 35 years’ experience as a thoracic Radiologist. R2 has 8 years and R3 has 2 years of experience as a thoracic Radiologist. R1 is a radiology resident in training to become a thoracic Radiologist. * Green: most frequent score.

0 was the most frequent score for pleural thickening (77%) and consolidation (70%); it was also the most frequent score in GGO (50%) and air-trapping (48%), albeit close to the frequency of +1 (35%, 33%, and 41%, respectively). A score of +1 was most frequent in peripheral airways (47%), micronodules (65%), and reticulations (58%) (Figure 5). A score of +2 was infrequent and only seen in peripheral airways (3%), micronodules (4%), and air-trapping (1%).

Highest cumulative scores were seen in micronodules (69%), reticulations (58%), and peripheral airways (51%).

A score of −1 was encountered in all structures/abnormalities except consolidation and most frequently in GGO (15%) and air-trapping (18%). A score of −2 was never scored in this group.

### Qualitative assessment - overall image quality

For both IQ100 and IQ40, the median VGA score for all readers was significantly better compared to EIDCT (median > 0; *p* < 0.0001). The most frequent VGA score for overall image quality was +1 in the IQ100 (78%) and 0 in the IQ40 group (56%). This is in line with the findings for all structures/abnormalities combined, where IQ100 shows a median and most frequent score of +1, and IQ40 shows a median and most frequent score of 0. The cumulative positive score (≥+1) for overall image quality was 84% in IQ100 and 41% in IQ40.

### Qualitative assessment - interobserver agreement

Interobserver agreement was fair (0.300). Detailed information of the scoring per reader is shown in Table 4. For IQ100, a score of +1 was the most frequent score for reader 1-3 (R1-R2-R3). For reader 4 (R4), score 0 was more frequently given. R1-R3 also gave the least amount of −1 and −2 scores, compared to R4.

**Table 4:** Summary of Reader Score

The most experienced reader was R4 with 35 years’ experience as a thoracic Radiologist. R2 has 8 years and R3 has 2 years of experience as a thoracic Radiologist. R1 is a radiology resident in training to become a thoracic Radiologist. * Green: most frequent score.

## Discussion

In our study, we did a side-by-side comparison between UHR-PCCT and HR-EIDCT at both a normal dose setting (PCCT-IQ100) and lower radiation dose settings (PCCT-IQ40). We found a significantly better image quality for PCCT-IQ100 compared to EIDCT. Using PCCT-IQ40, we were able to significantly further reduce radiation dose compared to EIDCT, with an image quality non-inferior to EIDCT.

In PCCT-IQ100, the significant better image quality was especially true for peripheral airways, micronodules, and reticulations (cumulative positive scores >75%). Better detection of these fine structures on PCCT could potentially have significant impact on the diagnosis of early findings of ILD and post-transplant complications. Indeed, a recent study showed promise for better visibility of ILD-related findings, such as reticulations, and showed a diagnostic impact in a small amount of patients with ILD (4 of 112 patients).^20^ In our population, reticulations and traction bronchi(ol)ectasis are much less common but still very important to detect, for example in the setting of early RAS.

Although PCCT showed significantly better visibility compared to EIDCT for all structures in IQ100 and for some in IQ40 (peripheral airways, micronodules, and overall image quality), the median VGA score for both IQ100 and IQ40 was also less than +2 (median = 2; *p* *≥* 0.016). This means that the improved image quality was not perceived to have a significant impact on diagnostic interpretation in our study population. For example, reticulations were more clearly visible on PCCT but were also detectable on EIDCT in most patients.

The improved image quality in PCCT-IQ100 was less apparent for air-trapping (highest cumulative score of 15%). Due to differences in expiration depth or motion artefacts, air-trapping can be difficult to compare between different scans. Still, air-trapping was found in the radiology reports of 69% of our cohort and represents the development of BOS in many of these patients. Median score for air-trapping was 0 for both PCCT-IQ100 and PCCT-IQ40, and a VGA score of −1 was primarily found in the evaluation of air-trapping and much less for the other structures/abnormalities (IQ100 = 15%; IQ40 = 18%); this suggests that although PCCT is overall non-inferior to EIDCT, in individual cases air-trapping can actually be more difficult to evaluate on PCCT than on EIDCT (Figure 4). Since air-trapping is important in evaluating BOS during long-term follow-up of lung transplant patients,^15^ the image quality of PCCT in evaluating air-trapping should be further evaluated in other studies.

Radiation dose was reduced by 33% (DLP) in PCCT-IQ100 vs EIDCT. This is in line with previous reports suggesting a dose reduction of 32% is feasible in patients with ILD.^20^ Although this is already a large radiation dose reduction compared to EIDCT, using the PCCT-IQ40 protocol radiation dose was even reduced by 73% compared to EIDCT. This significant further radiation dose reduction is especially beneficial as these are patients that require regular and long-term follow-up with imaging, preferably HRCT. The average age at transplantation in our population was 50 years, but the youngest patient included was only 20 years old. With yearly HRCT, radiation dose can potentially accumulate above desirable levels.^16^

This study has some limitations. First, a possible weakness in our study is the subjective nature of our scoring system. However, subjective reader scoring, using the Likert scale and other scales, has been used in several recent studies evaluating the impact of PCCT in imaging of the lung,^6^, ^20^ and is considered a way to evaluate the performance of PCCT in real-life practice. Also, previous studies have shown that objective noise is not well correlated with image quality.^20^ In our study, the GNL was better for IQ100 than IQ40 and non-inferior to EIDCT in both groups.

Our comparison was unblinded, which may cause bias. Further studies could benefit from blinded comparison and objective detection analysis. However, in our study, due to the high contrast-to-noise ratio of PCCT that makes the images highly recognizable, blinded comparison was deemed not feasible.

Second, our interobserver variation score is only fair (0.300). Interestingly, we see overall more neutral to lower scores (score 0 >+1, and more often −1) the more experienced the reader (Table 4). We could speculate that more experienced colleagues benefit less from increased resolution for their diagnostic decision-making because of an already higher sensitivity, specificity, and diagnostic confidence, similar to studies reporting that artificial intelligence (AI) systems that aid in chest radiograph interpretation have more impact on the detection and interpretation time for residents and fellow compared to experience thoracic radiologists.^21^ Still, the higher interobserver variability limits our generalizability and should be studied further. It also highlights the need for standardized reporting by radiologists experienced in lung transplant follow-up.

Future studies are needed to evaluate the clinical impact of PCCT, which remains currently unclear. The use of CT in general (when available) for follow-up of lung transplant patients is considered valuable to detect CLAD, especially RAS, and is part of the ISHLT diagnostic re--ations for follow-up and RAS diagnosis.^9^ Doing this at a significantly reduced radiation dose using PCCT not only reduces life-time radiation-related risks but also lowers the threshold to implement CT follow-up in centers that currently do not use HRCT in the follow-up of lung transplant patients. Whether or not the improved image quality of PCCT has an additional benefit in detecting abnormalities remains to be clarified, as we found no significant impact on diagnosis.

## Conclusion

Although image quality was scored significantly better in PCCT-IQ100, the image quality of PCCT-IQ40 was still adequate (non-inferior to EIDCT), and the significant radiation dose reduction of this protocol can greatly benefit this subset of often-young patients requiring lifelong, regular follow-up after lung transplantation. With future developments in PCCT, the improved image quality could have more impact on the detection of subtle findings: e.g., earlier detection of fine reticulations in RAS or of airways abnormalities in BOS. On the other hand, further radiation dose reduction is also likely in the future. In both scenarios, special attention is needed to safeguard the quality of expiratory scans and the visibility of air-trapping in PCCT.

## Financial support

The author and co-authors have no relevant disclosures. This study has received no grants or funding.

## Conflicts of Interest statement

The authors declare that they have no known competing financial interests or personal relationships that could have appeared to influence the work reported in this paper.

## Appendix A Supplementary material

*Table * - Summary of absolute score ** supplemental material. Summary of absolute VGA scores per structure/abnormality and for overall image quality, showing most frequent scores of 0 and +1 for IQ100 and IQ40. * Dark green: most frequent score. Lighter green: second most frequent score

.

*Figure * - GNL boxplots for lung tissue, soft tissue, and fat tissue ** supplemental material*.* Global noise levels (GNL), represented as boxplots, show a lower GNL for PCCT-IQ100 for lung tissue (a), soft tissue (b) and fatty tissue (c). The GNL of PCCT-IQ40 is closer to that of EIDCT

## Footnotes

Appendix A Supplementary data associated with this article can be found in the online version at doi:10.1016/j.jhlto.2026.100547.
