# Supplementary figures and images for: Ultra-high resolution photon-counting CT of the lung after lung transplantation: should we go for optimal image quality or reduced radiation dose?
Source: JHLT Open. 2026 Apr 11;13:100547. doi: 10.1016/j.jhlto.2026.100547 (PMC13141548; doi:10.1016/j.jhlto.2026.100547)

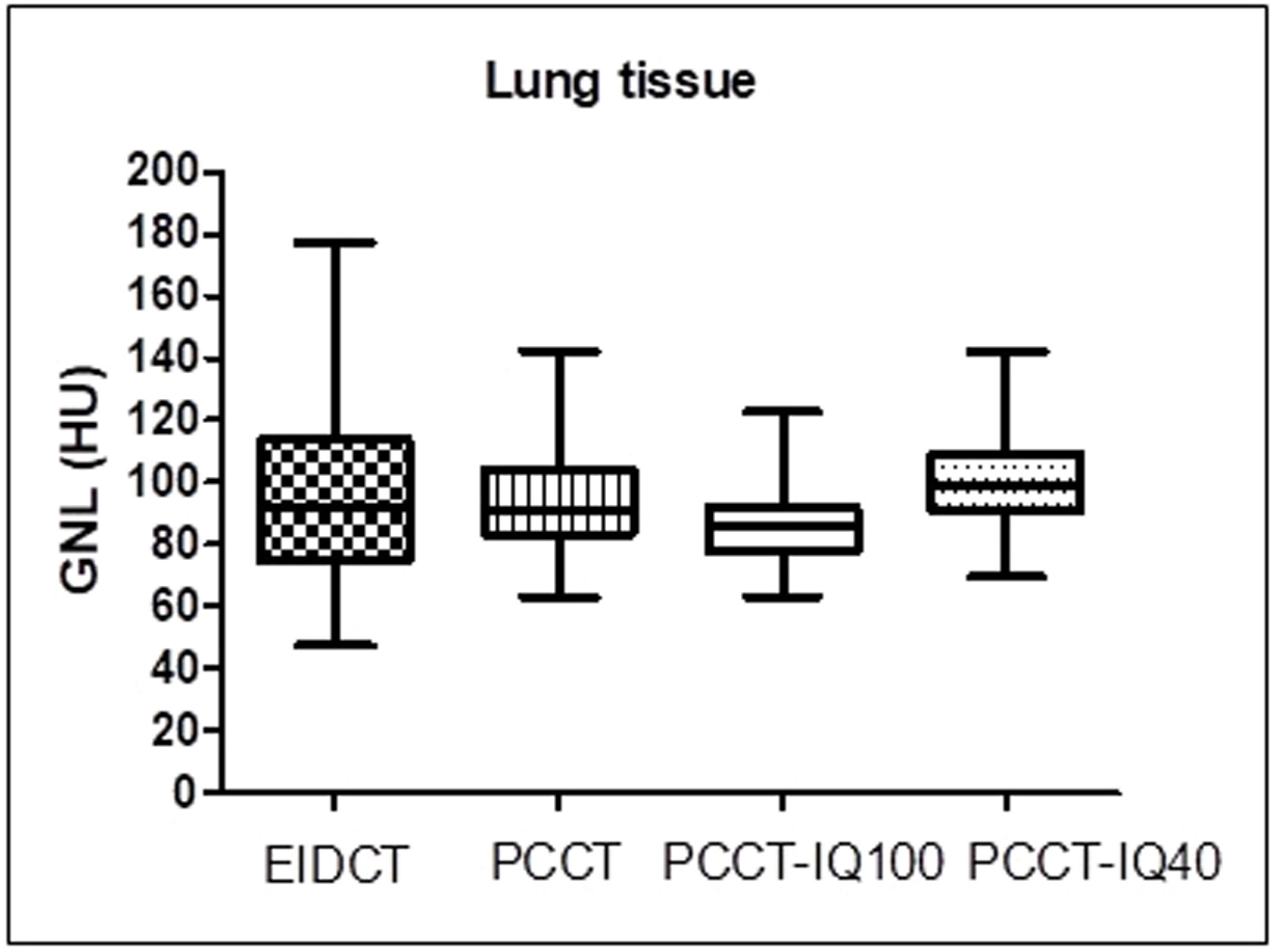

Supplement: Supplementary file 2 — Figure * - GNL boxplots for lung tissue, soft tissue, and fat tissue * supplemental material. Global noise levels (GNL), represented as boxplots, show a lower GNL for PCCT-IQ100 for lung tissue (a), soft tissue (b) and fatty tissue (c). The GNL of PCCT-IQ40 is closer to that of EIDCT [file mmc2.jpg]

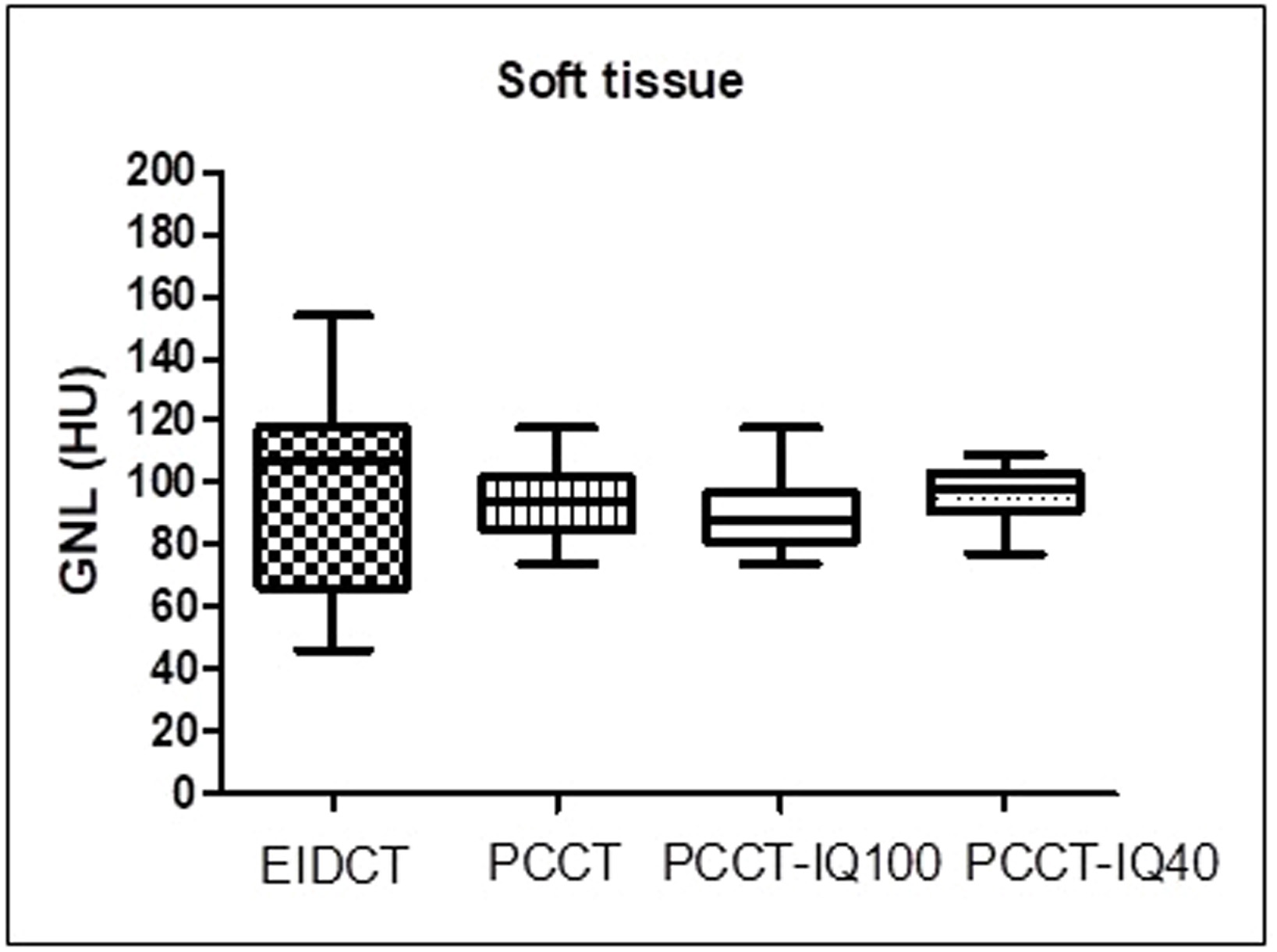

Supplement: Supplementary file 3 — Supplementary material [file mmc3.jpg]

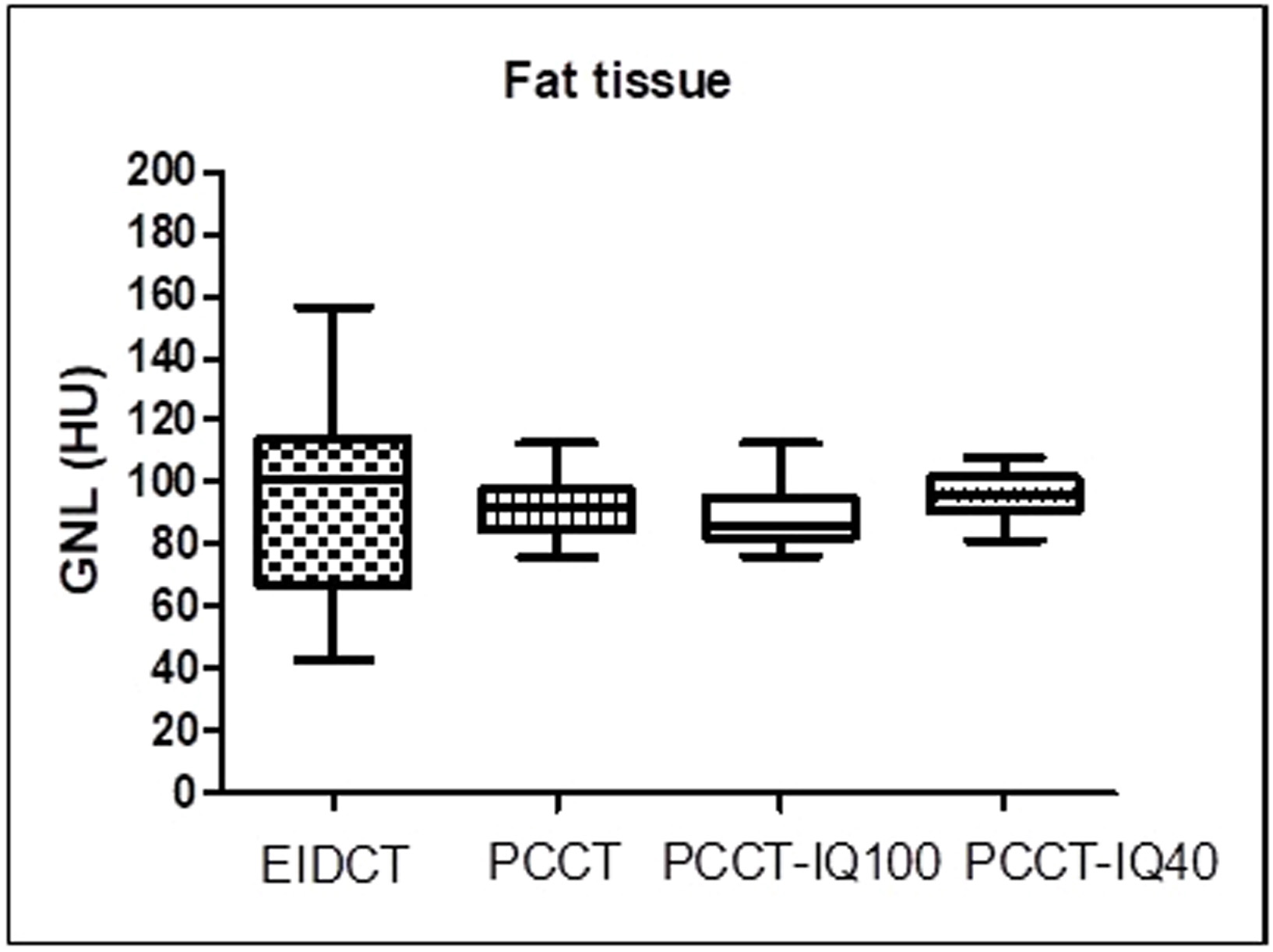

Supplement: Supplementary file 4 — Supplementary material [file mmc4.jpg]
